# Editorial: Hepatic immune response underlying liver cirrhosis and portal hypertension

**DOI:** 10.3389/fimmu.2023.1174562

**Published:** 2023-03-09

**Authors:** Yangkun Guo, Xiong Ma, Yongzhan Nie, Enis Kostallari, Jinhang Gao

**Affiliations:** ^1^Lab of Gastroenterology and Hepatology, West China Hospital, Sichuan University, Chengdu, China; ^2^Division of Gastroenterology and Hepatology, National Healthy Commission (NHC) Key Laboratory of Digestive Diseases, State Key Laboratory for Oncogenes and Related Genes, Renji Hospital, School of Medicine, Shanghai JiaoTong University, Shanghai Institute of Digestive Disease, Shanghai, China; ^3^State Key Laboratory of Cancer Biology, National Clinical Research Center for Digestive Diseases and Xijing Hospital of Digestive Diseases, Air Force Military Medical University, Xi’an, China; ^4^Division of Gastroenterology and Hepatology, Mayo Clinic, Rochester, MN, United States

**Keywords:** liver cells, immune response, liver fibrosis, portal hypertension, gut-liver axis

## Introduction

Under various etiological stimulations, such as alcohol, viruses, Western diet, endotoxins from gut microbiota, or circulating antigens, hepatic immune homeostasis is disrupted leading to chronic liver diseases and eventually liver cirrhosis (1; 2; Gan et al.; 3). Immune cells are recruited and activated at the sites of liver injury, regulating the local microenvironment and the progression of liver diseases (4) (Lan et al.). Although prominent efforts have been made in hepatic immunity and liver cirrhosis, the worldwide morbidity and mortality of liver cirrhosis remain high. Portal hypertension is the leading cause of cirrhosis-related death (5, 6). However, the molecular and cellular mechanisms underlying the hepatic immune response during liver fibrosis/cirrhosis and portal hypertension remain unclear. This Research Topic consists of 18 articles that present recent advances in uncovering the immune mechanisms underlying liver cirrhosis and portal hypertension. These investigations and reviews mainly focus on immune homeostasis and gut microenvironment in liver cirrhosis, providing potential new therapeutic strategies to treat liver cirrhosis and portal hypertension.

## Liver cells and the hepatic immune response during injury

Liver is composed of several cell types, mainly including hepatocytes, cholangiocytes, liver sinusoidal endothelial cells (LSECs), hepatic stellate cells (HSCs), and Kupffer cells (KCs). In case of injury, other immune cells such as monocyte-derived macrophages, Natural Killer (NK) cells, neutrophils, T cells, or B cells are recruited to the liver (7, 8). Following liver injury, infiltrated immune cells release proinflammatory cytokines, and eventually mediate HSC activation and extracellular matrix (ECM) deposition (Gan et al.). In this Research Topic, original research papers and reviews will demonstrate and comment on the role of KCs, macrophages, T cells, and neutrophils in the pathogenesis of liver fibrosis/cirrhosis, as well as how immunity-related genes (IRGs) dominate immune cell infiltration and chronic inflammatory reactions in the liver.

### KCs and monocyte-derived macrophages

KCs are liver resident macrophages that are generated during embryonic period and adulthood (9, 10). Embryo-derived KCs (Em-KCs) are maintained in the liver throughout the life cycle through a self-renewal process (Li et al.). In adults, bone marrow (BM)-derived monocytes can migrate to the KC pool when Em-KCs are exhausted (Li et al.). KCs have been historically classified into proinflammatory M1 and pro-repairing M2 phenotypes. However, M1/M2 classification is not adapted to accurately identify KC subtypes during liver injuries. Recently, two clusters of KCs were identified in the murine livers by single-cell RNA sequencing (scRNA-seq). KC1 (major sub-population, cluster of differentiation (CD) 206^lo^ESAM^-^) that possesses tolerogenic immune responses, and KC2 (minor sub-population, CD206^hi^ESAM^+^) that is characterized by a proinflammatory and metabolic profile (Gao et al.). Only a small amount of monocyte-derived macrophages resides in the liver in homeostasis (11, 12). Hepatic damage promotes monocyte-derived macrophage accumulation to the liver (13). A broad-spectrum of macrophage activation states is revealed by scRNA-seq and cellular indexing of transcriptomes and epitopes by sequencing (CITE-seq) data in human fibrotic livers (Gao et al.). In addition, the different sub-populations of macrophages/KCs were identified utilizing markers, such CD163, macrophage receptor with collagenous structure (MARCO), and V-set and immunoglobulin domain containing 4 (VSIG4) (Li et al.). Further investigations are needed to better understand the role of each subpopulation during liver diseases.

Functionally, C-C motif chemokine receptor 2 (CCR2)^+^ and CCR5^+^ macrophage infiltration in murine livers exacerbates alcohol-associated liver disease (ALD) progression (Xu et al.). Moreover, activated KCs/macrophages increase portal pressure by inducing the release of vasoconstrictors, and promote liver fibrosis by enhancing HSC transdifferentiation into fibroblast-like cells (Li et al.). In addition to cell crosstalks, endoplasmic reticulum (ER) stress also enhances the metabolic re-programming and activation of KCs and macrophages (Zhou et al.). Finally, macrophage-specific c-Jun N-terminal kinase (JNK), nuclear factor kappa-B (NFκB), Jauns kinase (JAK)- signal transducer and activatior of transcription (STAT), and Notch signaling pathways contribute to the inflammatory response and liver fibrosis progression. On the opposite, activation of Wnt/β-Catenin signaling pathway in macrophages promotes the resolution of liver fibrosis (Gao et al.). In summary, targeting KCs/macrophages might provide a novel therapeutic strategy for liver fibrosis.

### T cells

T cell family includes tissue-resident memory T (T_RM_), CD4^+^, CD8^+^, and γβ T cells, originating from naïve T-cell precursors and presenting a pro- or anti-fibrotic role in the liver (14, 15) (Zhang and Zhang). The growth, proliferation, and differentiation of liver T_RM_ cells are mediated by cytokines such as interleukin (IL)-2, IL-15, IL-10 and transforming growth factor-β (TGF-β) (Li et al.). Hepatic T_RM_ cells play a significant anti-infection role in chronic viral hepatitis (Li et al.). In nonalcoholic fatty liver disease (NAFLD), the number of liver T_RM_ cells positively correlates with systemic inflammation in patients with obesity. However, a novel subset of T_RM_ cells (CD69^+^CD103^-^CD8^+^) shows a protective function in NASH-related fibrosis (Li et al.). Thus, hepatic T_RM_ cells might serve as a novel immunotherapy strategy for chronic liver diseases.

Based on scRNA-seq studies, mice with nonalcoholic steatohepatitis (NASH) present an accumulation of CD4^+^, CD8^+^, and γβ T cells in the liver (16). Recently, it has been shown that activated CD4^+^ T cells contribute to the progression of NASH-related inflammation and fibrosis (Zhang et al.). Although initial investigations have started to elucidate the role of T cells in the pathogenesis of liver disease, further studies are needed to explore the heterogeneity as well as their interaction with other liver cells.

### Neutrophils

In a healthy liver, there are very few resident neutrophils. However, in case of a pathogen invasion, neutrophils from the circulation migrate into the liver (17). Neutrophil infiltration into the liver during ALD correlates with the upregulation of the glycoprotein lipocalin (LCN2) on neutrophils (Xu et al.). Moreover, patients with ALD exhibit a deficient AKT/p38-MAPK signaling, myeloperoxidase release and bactericidal activity (Xu et al.). The recruited neutrophils are involved in the innate immune response during NASH-related fibrosis as well (18, 19). Furthermore, activation of the inositol-requiring enzyme 1 (IRE1α)- X box binding protein-1 (XBP1) signaling pathway stimulates neutrophil differentiation (Zhou et al.). In summary, neutrophils are critical immune cells involved in the development of chronic liver diseases.

### LSECs

LSECs are the most abundant nonparenchymal cells in the liver and are the gatekeepers of the liver microenvironment (20). The disrupted intercellular crosstalks between LSECs and other cell types within the sinusoids are involved in the pathogenesis of liver fibrosis (Du and Wang). Vascular cell adhesion molecule 1 (VCAM1) endothelial-specific deletion attenuates macrophage accumulation in the liver and hepatic fibrosis (Guo et al.). Thus, restoring the crosstalk between LSECs and other liver cell types by targeting adhesion-related molecules, NO-related signaling pathways, and angiogenesis may serve as effective therapeutic strategies for liver fibrosis (Du and Wang). Additional studies are important to uncover other signaling pathways involved in the crosstalk between LSECs and other liver cell types to better understand the pathobiology of liver diseases.

### Other immune cells and immune-related genes

Liver fibrosis is associated with altered hepatic immune response (Zhou et al.). Toll-like receptor (TLR) expression and activity increase aggravates liver fibrosis during the co-occurrence of NAFLD and HBV infection *via* enhancing the infiltration and activation of adaptive immune cells, such as CD8^+^ T cells and NKT cells (Tourkochristou et al.). By analyzing liver scRNA-seq (GSE136103) and RNA microarray (GSE45050) datasets from patients with cirrhosis, Liu et al. showed that four immunity-related genes in NK cells, including interferon regulatory factor 8 (*IRF8*), nuclear receptor subfamily 4 group A member 2 (*NR4A2*), IKAROS family zinc finger 3 (*IKZF3*), and *REL*, are involved in liver fibrogenesis. (Liu et al.). Additionally, the peroxisome proliferator-activated receptor (PPAR) has a therapeutic potential for treating primary biliary cholangitis (PBC) (Wang et al.).

Systemic vasculitis is an autoimmune disease characterized by increased vascular wall inflammation and necrosis. Patients with systemic vasculitis can be treated with immunosuppression therapies (21). However, when immunosuppression is ineffective, portal venous angioplasty followed by stent placement may be an alternative strategy to prevent portal hypertension-related complications (Cai et al.). Further clinical investigations are needed to clarify the efficacy of the above targets and therapeutic strategies in the clinical treatment of liver diseases.

## The contribution of gut-liver axis to liver diseases

Hepatic immunity is affected not only by liver damage but also by other organs, including the gut, spleen, lung, brain, and adipose tissue (Zhang et al.). The dysfunctional gut-liver axis leads to a “leaky gut”, which relates to bacteria’s toxic metabolites infiltrating into the circulation and the liver, leading to macrophage and neutrophil accumulation and subsequent liver fibrosis progression (Guan et al.) (22–24). Recently, an increasing number of studies have shown that the intestinal flora is involved in the pathogenesis of NAFLD by affecting metabolism, intestinal endotoxin, and intestinal mucosal permeability (Liu et al.). Moreover, increased portal vein pressure causes intestinal edema and decreases intestinal motility, terminally changing gut microbial diversity (25). Splenectomy could significantly reduce portal hypertension (26). Consistently, restoring gut microbiome by splenectomy in addition to pericardial devascularization improves liver function and reduces intestinal permeability (Zhao et al.). Likewise, injecting gut microbial metabolite, trimethylamine-N-oxide (TMAO), also restores the integrity of endothelium in a NASH-associated fibrosis model (Zhang et al.).

Sex and sex-related hormones also play a crucial role on gut microbiota diversity (Xu et al.). Since androgen-induced dysbacteriosis makes males more vulnerable to metabolic imbalance than females, hormones also provide a safe and effective way of treating liver diseases (Xu et al.). Other strategies, including fecal microbiota transplantation and antibiotic treatment, have been proposed to treat liver diseases by targeting the dysfunctional gut-liver axis. However, increased risks of antibiotic resistance and pathogen infection have limited their clinical application in liver diseases (Liu et al.). In summary, the crosstalk between the liver and other organs contributes to hepatic homeostasis, which might provide complementary therapeutic strategies for liver cirrhosis.

## Conclusions

This Research Topic summarizes the current advances regarding hepatic immune response at the cellular and molecular levels. Specific subtypes of macrophages, T cells, neutrophils, and LSECs have started to be considered as potential targets for clinical treatment of liver fibrosis/cirrhosis. Although the field of understanding liver inflammation is progressing rapidly, more studies are needed to find novel therapies for liver cirrhosis.

## Author contributions

JG, EK, YN, and XM conceived and supervised the study; YG, JG, EK, YN, and XM wrote and revised the manuscript. All authors contributed to the article and approved the submitted version.
